# Microscopic resection of a giant epidermoid tumor of the pineal region via supracerebellar infra and transtentorial approach with endoscope assistance

**DOI:** 10.3171/2021.4.FOCVID2128

**Published:** 2021-07-01

**Authors:** Marco Antônio Zanini, Aderaldo Costa Alves Junior, Fabio Pires Botta, Haniel Moraes Serpa Nascimento, Pedro Tadao Hamamoto Filho

**Affiliations:** 1 Department of Neurology, Psychology and Psychiatry, Division of Neurosurgery, Botucatu Medical School, São Paulo State University, UNESP, Botucatu, São Paulo, Brazil; and; 2 Sunnybrook Health Sciences Centre, University of Toronto, Ontario, Canada

**Keywords:** pineal region tumor, epidermoid tumor, supracerebellar infratentorial approach, endoscope assistance

## Abstract

The authors present a case of a 22-year-old male who developed hydrocephalus symptoms related to a giant epidermoid tumor at the pineal region. The surgical approach and technique for a large epidermoid tumor in this area are extensively discussed. A paramedian contralateral supracerebellar infratentorial and transtentorial approach was performed, with the patient in a semisitting position. The tumor was removed using a microscopic technique, and endoscope assistance was used in order to reach the areas unable to be visualized under the microscope. The patient was neurologically intact at his 2-year follow-up, and postoperative MRI showed a significant decrease in the tumoral volume.

The video can be found here: https://stream.cadmore.media/r10.3171/2021.4.FOCVID2128.

## Transcript

### 0:26 Patient History

This is a case of a 22-year-old male with a history of a long-standing headache that worsened over the course of 3 months, associated with vomiting and decreased visual acuity, mainly on the right side.

A CT scan showed a hypodense tumor with similar density to CSF and no enhancement to contrast located in the pineal region leading to obstructive hydrocephalus. The patient underwent a ventricle-peritoneal shunt placement in a peripheral hospital and showed an improvement in his intracranial hypertension symptoms.

### 0:58 Neurological Examination

On admission to our service, he complained of dizziness and difficulty walking. A neurological examination showed only papilledema, and no focal deficits.

### 1:08 Preoperative Imaging Studies

T1 MRI showed a hypointense tumor measuring 4.8 × 6.2 cm with no enhancement to gadolinium. On T2 sequences the tumor was hyperintense. On FLAIR it exhibited some degree of heterogeneity but was predominantly hyperintense. The DWI showed restricted diffusion, favoring the diagnosis of an epidermoid tumor.

The tumor was centered at the pineal region, located on both the supra- and infratentorial compartments. Inferiorly, it occupied the quadrigeminal cistern, compressing the cerebellar culmen and the quadrigeminal plate, leading to the obstruction of the cerebral aqueduct. Superiorly, it extended until the splenium of the corpus callosum and toward the right thalamus and the atrium of the lateral ventricle, displacing these structures upward and laterally.

### 2:07 Surgical Approach Selection

There are two main approaches that are the most suitable for pineal region tumors, both with advantages and disadvantages: occipital interhemispheric transtentorial and supracerebellar infratentorial. The choice is based on the diagnosis, origin and projection of the tumor, as well as its size, location of the deep venous complex, the tentorial angle, and surgeon’s preference.

In this case, the large tumoral volume slightly extending to the right thalamus and atrium could cause difficulty in reaching the entire tumor circumference with any midline approach. To overcome this situation, we opted to perform a paramedian contralateral supracerebellar infratentorial approach in a semisitting position. This could allow for the removal of most of the midline mass, as the gravity would also assist in exposing more tumoral tissue for resection. A right tentorium opening could expand the view laterally, adding a transtentorial approach. Finally, the addition of an endoscopic view would permit the maximum visualization to the points where the straight microscopic light cannot reach.

### 3:22 Patient Positioning

The patient was placed in a semisitting position. The head was fixed with Mayfield pins and placed with a slight neck semiflexion, sufficient for a good exposure while keeping the airway open and maintaining unrestricted cervical venous drainage. Intraoperative expired carbon dioxide measurements were continuously monitored during the anesthetic procedure.

### 3:45 Incision

A midline incision 5 cm above the inion extending to the C3 spinous process was marked, and a suboccipital craniotomy with slight left predominance was planned, in order to aim a left paramedian approach directed to the right-sided tumor.

### 4:02 Craniotomy and Dural Opening

The extended suboccipital craniotomy exposed the sinuses’ confluence in such a way that a supratentorial approach could be performed if necessary. The dura was opened in a “Y” shape, elevating the superior edge to improve the corridor vision. Small dural adherences and bridging veins were cut, allowing for the cerebellum to naturally fall, providing the surgical path to the pineal region.

### 4:27 Tumor Visualization and Dissection

After opening the arachnoid, the typical pearly aspect was visualized and the removal started in a piecemeal fashion. Dissection and aspiration could be easily performed, gaining space within the quadrigeminal cistern.

### 4:43 Tumor Resection

Portions of the tumor capsule were carefully removed, decompressing the posterior portions of the brainstem. From a more superior view, tumor located around the venous complex continued to be removed. To enhance the superior view, more cisternal opening was performed toward the apex of the tentorial notch. Next, dissection of the inferior portion of the tumor capsule was done in an anterior, superior, and lateral direction.

### 5:21 Endoscope Assistance

At this point, when the microscopic view became limited, the neuroendoscope was used to take advantage of its flexibility and allow the continuation of the tumor removal.

The endoscopic view showed a large portion of the tumor hidden above the tentorium to the right, so a tentorial opening was planned on the right side.

### 5:37 Tentorium Opening

Under microscope vision, the tentorium was split using an 11 blade and microscissors, giving special attention to the free edge of tentorium to identify and not damage the fourth cranial nerve.

After visualizing and moving the right trochlear nerve, the tentorium was fully sectioned, expanding the workspace for the supratentorial portion of the tumor.

### 6:03 Tumor Resection

Moderate-pressure saline jets also helped the tumor removal by mobilizing the free tumor parts.

After removing large portions of the tumor, the superior aspect of the capsule could be visualized and removed with the assistance of endoscopic view. The adherences of the tumor to neural and vascular structures demanded meticulous dissection technique.

### 6:28 Endoscopic Tumor Removal

With a lateral rotation of the endoscope view, it was possible to visualize and remove more supratentorial tumor. The trochlear nerve was in the forefront, and it was always kept in the field to avoid inadvertent damage.

By working on both medial and lateral spaces to the fourth cranial nerve, more of the tumor and its capsule continued to be removed.

### 6:54 Microscopic Tumor Removal

Again, under microscopic view, the remainder of the capsule and tumor debris were removed. Progressively, the anterior, superior, and lateral limits of the tumor became more apparent.

### 7:09 Hemostasis

Careful hemostasis was performed throughout all the surgical procedure, particularly after the end of the tumor resection. Parts of the capsule that were firmly adhered to neural and vascular structures were left in place to avoid major risks of brain damage.

### 7:24 Final Endoscopic Inspection

The endoscope was used once more for final inspection, showing the tumoral site with some debris and remnants of the capsule. Through the tentorial incision, parts of the supratentorial compartments were seen, including the posterior aspect of the parahippocampal gyrus.

### 7:42 Closure

At the end of the procedure, the right trochlear nerve could be seen intact and the closure was performed in a standard layered fashion.

### 7:51 Postoperative

After the surgery, the patient did not develop any neurological deficits. In a 2-year follow-up, he continued to be asymptomatic, and a postoperative MRI showed a significant decrease in the tumoral volume.

### 8:08 Discussion

Epidermoid tumors, also named epidermoid cysts, are benign lesions of slow growth. They are considered to be originated from ectodermal cells that were trapped within the central nervous system during the 3rd and 4th weeks of intrauterine development.^1^ They are most commonly found at the cerebellopontine angle, middle cranial fossa, fourth ventricle, and chiasmal region.^2^ Pineal region epidermoid tumors account for 3%–4% of all intracranial epidermoid tumors and for 3.4% of all pineal region tumors.^3^

Epidermoid tumors in this location can be managed either by the occipital interhemispheric transtentorial approach or by the supracerebellar infratentorial approach.^1^ The endoscope assistance is helpful for increasing visibility, illumination, and maneuverability of instruments in a tight surgical corridor, and it can even be used as the sole option for tumors in the pineal region.^4^ The upward displacement of venous structures favors the use of the supracerebellar infratentorial approach; however, larger tumors with supratentorial extension may require other strategies, such as the combined use of a transtentorial approach, as was done in the present case.

Tumor remnants are acceptable in cases of tight adherence to neurovascular structures, and the patients should be closely monitored to detect regrowth.^1,2,5^

## Acknowledgments

We give special thanks to Michael Koenen, RN, for providing the voice-over for this video.

## Author Contributions

Primary surgeon: Zanini. Assistant surgeon: Costa Alves Junior, Botta, Moraes Serpa Nascimento. Editing and drafting the video and abstract: Costa Alves Junior, Botta, Tadao Hamamoto Filho. Critically revising the work: Costa Alves Junior, Zanini, Tadao Hamamoto Filho. Reviewed submitted version of the work: Costa Alves Junior, Tadao Hamamoto Filho. Approved the final version of the work on behalf of all authors: Costa Alves Junior. Supervision: Zanini, Tadao Hamamoto Filho.
